# Preschool Teachers’ Technology Acceptance During the COVID-19: An Adapted Technology Acceptance Model

**DOI:** 10.3389/fpsyg.2021.691492

**Published:** 2021-06-07

**Authors:** Xiumin Hong, Mingzhu Zhang, Qianqian Liu

**Affiliations:** Institute of Early Childhood Education, Faculty of Education, Beijing Normal University, Beijing, China

**Keywords:** preschool teachers, technology acceptance model, behavioral intention, determinant factors, COVID-19

## Abstract

Online education has become a major reaction to the COVID-19 epidemic, which requires preschool teachers to quickly adapt to online education and accept educational technology. In this emergency background, research on the preschool teachers’ technology acceptance provides clues to improve preschool teachers’ intention to use educational technology. The Technology Acceptance Model (TAM) is widely used to examine the process of individuals’ technology acceptance in the previous literature. Therefore, this study seeks to examine preschool teachers’ technology acceptance with the adapted TAM and their determinant factors. The proposed model was empirically validated by using survey data from 1,568 preschool teachers during the COVID-19. Results indicate that preschool teachers’ behavioral intention was moderate to high level. Perceived usefulness and perceived ease of use are direct significant predictors of preschool teachers’ behavioral intention. Perceived usefulness is affected by perceived ease of use and job relevance. Computer self-efficacy and perceptions of external control are the positive factors toward perceived ease of use. Our findings present powerful evidence for the applicability of the adapted TAM in a sample of Chinese preschool teachers under emergency circumstances. These results highlighted some potential avenues for interventions aimed at improving preschool teachers’ acceptance toward educational technology.

## Introduction

During the COVID-19, the shift from the offline education to emergency distance teaching has made teachers feel unprecedented pressure to use technology (Ferdig et al., 2020; Knig et al., 2020; Quezada et al., 2020). Preschool teachers are no exception. They interact with young children and their families, share text and video resources, participate in remote training and meetings, and deal with daily work affairs by computer-based educational technology, which they hardly ever experienced. Therefore, this study believes that with the swift transition to distance teaching, Chinese preschool teachers may face unprecedented challenges in technology acceptance. The application of educational technology, which is critical to the success of online education during the COVID-19, aims to reduce face-to-face courses and enforce social distancing. However, such expectations come to nothing if preschool teachers lack behavioral intention to use technology, because any technology or system’s success depends on how it is used by its target users (Granić and Marangunić, 2019). Research examining technology acceptance has generally focused on the students and pre-service teachers, however, to the authors’ knowledge, there have been no empirical studies to determine the preschool teachers’ technology acceptance intention and the influential factors, especially in the context of a global public health emergency. As such, the authors argue that it is crucial to examine preschool teachers’ intentions toward computer-based educational technology and the determinants.

The Technology Acceptance Model (TAM) and extended TAM have been broadly applied to the technology acceptance field in order to ascertain the pivotal determinants (Joo et al., 2018b; Rafique et al., 2018). Based on the TAM, our study’s main purpose is to explore preschool teachers’ acceptance toward computer-mediated educational technology and key determinant factors during the COVID-19. More specifically, the present study would be guided by the two research questions: How is the preschool teachers’ acceptance intention to educational technology during the COVID-19? What are relationships between preschool teachers’ technology acceptance intention and its determinant factors during the COVID-19?

## Literature Review

This part first presents preschool teachers’ educational technology usage during the COVID-19. Secondly, we introduce in detail the theoretical model, namely TAM, to explore the preschool teachers’ technology acceptance and its influential factors.

### Preschool Teachers’ Educational Technology Usage During the COVID-19

Educational technology has become an important weapon in the education sector during the COVID-19 epidemic (Iivari et al., 2020). Many countries have taken the measures of switching to online education to reduce the harm of coronavirus (Jandrić, 2020; OECD, 2020; Zhang et al., 2020). Like many countries around the world, all preschools in China were forced to switch to online education in the spring semester of 2020 as a response to epidemic prevention. As such, all preschool teachers were required to quickly and skillfully accept and use educational techniques they were not familiar with before (Hodges et al., 2020; Rapanta et al., 2020). This development requires preschool teachers to meaningfully accept and use technology in the online teaching environment. This accepting and using includes, but is not limited to, connecting people (i.e., children, parents, colleagues, and leaders), integrating resources, and collaborating through educational technology (von Davier et al., 2017). For preschool teachers, they need to carefully select resources suitable for young children’s learning and parent-child interaction, produce audio and video clips, and answer parents’ parenting questions, which all depend on educational technology.

Teachers’ willingness and acceptance toward technology play a crucial role in the successful application of educational technology (Yuen and Ma, 2008). For a long time, Chinese preschool teachers lack the ability to use educational technology. Preschool Teachers’ Professional Standards (Ministry of Education, 2012), a national professional standard, has not made definite requirements on Chinese preschool teachers’ information technology ability. In addition, online education was not widely conducted in preschool education in China before the COVID-19 (Hong, 2020). Only some preschool teachers communicate with families through the mobile phone or computer, but this is not for children’s mobile learning. However, preschool teachers rely on computer-mediated educational technology to deliver online education during the COVID-19. As such, Chinese preschool teachers may face challenges when receiving and using the educational technology.

To ensure the smooth development of online preschool education during the epidemic, it is essential to explore preschool teachers’ willingness to accept educational technology and its influencing factors. So far, however, researchers have not paid much attention to this topic. Although preschools began re-opening in the Chinese sector and in other parts of the world, any prediction as to when the epidemic will be over and closures of preschools will end finally seems to be hardly complete at the moment. Hence, it is a growing necessity to explore and examine their behavior intention toward educational technology and why the technology is accepted or rejected. Our findings would provide insights into preschool teachers’ technology acceptance and use during the epidemic, which would help support preschool educational continuity.

### Technology Acceptance Model

The TAM is widely used to examine the process of individuals’ acceptance of technology. The TAM, arising from the theory of reasoned action (Madden et al., 1992), is developed by Davis (1989), and then extensively used in the technology acceptance field to explain technology use behavior (Zha et al., 2015; Yoon, 2016; Rafique et al., 2018; Teo et al., 2018). The TAM contains core components, i.e., use motivation, including perceived ease of use, perceived usefulness, and outcomes including behavioral intention. Guided by the TAM’ s viewpoint, people’s behavioral intentions to accept technology are impacted by the following crucial factors: perceived usefulness and perceived ease of use.

Behavioral intention means the degree of the individuals’ inclination and state of readiness before adopting technology behaviors (Ajzen, 1985). Perceived usefulness and perceived ease of use are taken as the most significant variables predicating behavioral intention in a direct or indirect way (Marangunić and Granić, 2015; Liu et al., 2019). Perceived usefulness is thought to be how much individuals believe that applying technology will promote their work outcomes (Davis, 1989). That is, if preschool teachers perceive advantages and serviceability when using educational technology (namely, perceived usefulness), their intention toward these technology resources and systems will be stronger. Perceived ease of use is identified as how individuals perceive that applying the target technology is free from effort to enhance achievement at work (Davis, 1989). Meanwhile, when educational technology is easy to understand and use (i.e., perceived ease of use), individuals will have a positive inclination toward the use of technology. Empirical research on the TAM explored the significant impact of perceived usefulness and perceived ease of use on users’ using intention toward technology (Valdehita et al., 2019; Rafique et al., 2020). Especially, in educational settings, teachers’ perceived usefulness and perceived ease of use are positively related to their acceptance intention to educational technology in teaching practice (Pynoo et al., 2012; Scherer et al., 2019). Therefore, this study proposes the following hypothesis:

Hypothesis 1: Perceived usefulness will have a positive significant impact upon the behavioral intention to use educational technology.
Hypothesis 2: Perceived ease of use will have a positive significant impact upon the behavioral intention to use educational technology.

Furthermore, the relationship between perceived ease of use and perceived use has been described in preceding studies. For example, a study conducted by Rafique et al. (2018) indicated that perceived ease of use affects perceived usefulness, resulting in increased behavioral intention. When individuals perceive that using a certain technology would get rid of difficulties or does not require a huge effort, the perception of this technology will be more helpful and useful. The following hypothesis was thus proposed for this construct:

Hypothesis 3: Perceived ease of use can positively affect the perceived usefulness of educational technology.

Precious studies replicated and validated the TAM in many fields, including the education sector. The TAM was mainly employed to examine individuals’ usage intention about developed tools and technologies, for example, exploring people’s acceptance of mobile libraries and electronic learning (Jeong, 2011; Chang et al., 2017) or to examine students’ acceptance of virtual laboratories, machine translation in education, YouTube (i.e., Lee and Lento, 2013; Valdehita et al., 2019; Yang and Wang, 2019). Besides, the TAM was employed to understand teachers’ educational technology acceptance behavior in teaching practices. Studies conducted in the sample of pre-service teachers and secondary school teachers revealed that the TAM’s validity in an educational context was applicable (Pynoo et al., 2011; Teo and Noyes, 2014). However, the background of the above research is mainly focused on higher education fields (Chang et al., 2017; Yang and Wang, 2019), and there are few studies on preschool education.

Although considerable evidence for the TAM can be found in studies with western teacher participants (Holden and Rada, 2011; Kusano et al., 2013; Perkmen et al., 2016), research with non-western teachers, still remains sparse to date. In addition, there were limited applications and replication of TAM in the education field, especially in developing countries. In China, a developing country in the East, the application and extension of TAM is limited and does not focus on preschool teacher (Teo et al., 2018).

### Development and Adaptation of TAM

Although numerous studies confirmed TAM robustness (Awwad and Al-Majali, 2015; Aburagaga et al., 2020), it is necessary to supplement several determining factors to TAM to explain comprehensively how individuals accept a certain technology (Nistor, 2014). Considering the demand, previous studies have developed and adapted TAM by adding various determinants, such as job relevance, computer self-efficacy, perceived of external control, and so on (Cheung and Vogel, 2013; Joo et al., 2018a; Yang and Wang, 2019).

The TAM presented by Davis holds that external variables (i.e., system design features) are associated with TAM constructs (Davis, 1989). Later, the studies for the TAM increased over time and some of them extended the TAM by additional external factors (e.g., job relevance, computer self-efficacy, and perception of external control) as determinants of TAM constructs (Schepers and Wetzels, 2007; Son et al., 2012; Ros et al., 2015). According to the TAM, perceived usefulness and perceived ease of use have different impact on individual acceptance tendency, which indicates that the unique role of these two antecedents in the process of individual technology acceptance should be investigated separately. Venkatesh and Bala (2008) proposed and explored the respective determinants of perceived usefulness and perceived ease of use. Job relevance is an antecedent variable of perceived usefulness, and perception of external control and computer self-efficacy are significant factors of perceived ease of use. Job relevance is recognized as to how individuals think technology is applicable to work (Son et al., 2012). Job relevance is positively associated with perceived usefulness, which is verified by relevant research (Sun et al., 2019). Perception of external control is how one perceives the existing organization and technology to support technology application (Ros et al., 2015). Researchers found that one’s perception about external resources and support is the primary feature affecting perceived ease of use (Ozturk et al., 2016; Alwabel et al., 2020). Besides, computer self-efficacy is defined as individuals’ speculation and judgment on whether he or she is capable of applying educational technologies (Celik and Yesilyurt, 2013). Previous studies suggested that perceived ease of use is significantly associated with beliefs, namely, computer self-efficacy (Scherer et al., 2015). In keeping with the literature, we proposed the following hypothesis:

Hypothesis 4: Job relevance will play a positively significant influence upon the perceived usefulness.
Hypothesis 5: Computer self-efficacy will be a significant predictor that positively affected the perceived ease of use toward educational technology.
Hypothesis 6: Perception of external control will be a positive factor that significantly impacted the perceived ease of use toward educational technology.

**FIGURE 1:**
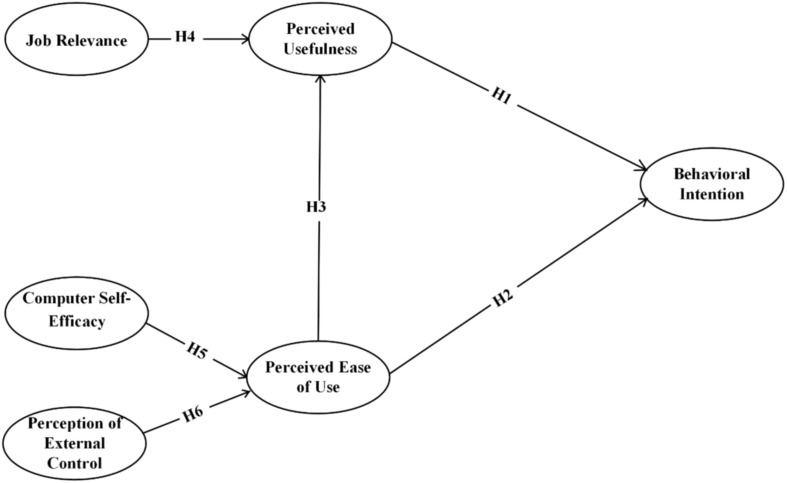
Descriptive statistics of study variables.

Although some previous studies have expanded and specified determinants of behavioral intention to use technology, to our best knowledge, little is known about the antecedent variables of education technology acceptance for preschool teachers. Constructing and testing a model with multiple influencing factors contributes to a more comprehensive understanding of the preschool teacher’s TBM. Motivated by prior literature, the current study employed the adapted TAM to construct the theoretical model in understanding preschool teachers’ acceptance toward educational technology during the COVID-19. As discussed in existing technology acceptance studies, behavioral intention has a strong effect on users’ actual behavior (Szajna, 1996; Liu et al., 2010). Therefore, the work at hand selects behavioral intention as the outcome variable. Perceived ease of use and perceived usefulness are principal variables of individuals’ technology acceptance (Hao et al., 2017; Teo et al., 2017). Job relevance as one of the external factors was described as a firm association with perceived usefulness (Pituch and Lee, 2006). Similarly, computer self-efficacy and perception of external control play a positive effect upon the perceived ease of use (Abdullah and Ward, 2016). According to the TAM’s existing research, our research projected the adapted theoretical model (see Figure 1 for details).

### The Present Study

Review of the existing literature, we found that the TAM model is widely applicable, which provides a clear theoretical basis and empirical reference for this study. However, as mentioned above, existing studies still have the following limitations: (1) There are many studies based on samples in the West countries, but few empirical studies based on the Eastern samples. (2) Students and pre-service teachers, instead of preschool teachers are focused in prior research. (3) Most literature examined the TAM under the normal circumstances rather than the epidemic period. The rapid adoption of online teaching under the COVID-19 implies a requirement to understand the prerequisites affecting preschool teachers’ inclination to accept educational technology. Motivated by the call of technology into early childhood education, the current study aims to explore preschool teachers’ acceptance intention and its determinant factors toward educational technology based on the adapted TAM. Specifically, we have the following research questions:

1. How is the preschool teachers’ acceptance intention to educational technology during the COVID-19?
2. What are relationships between preschool teachers’ technology acceptance intention and its determinant factors (i.e., perceived usefulness, perceived ease of use, job relevance, computer self-efficacy, perceptions of external control) during the COVID-19?

## Materials and Methods

### Participants

Participants in the current study were 1,568 preschool teachers randomly recruited from the National Training Program for Preschool Teachers during the COVID-19 epidemic in May 2020 in China. The National Training Program for Preschool Teachers is sponsored by Ministry of Education of People’s Republic of China and serves all preschool teachers. All preschool teachers who have participated in online education during the COVID-19 and have used educational technology can participate in our study. Most teachers are from public preschools (73.6%), and 70.6% come from urban areas. Teachers’ years of experience were selected into shorter than 3 years (5.0%), 3–5 years (10.7%), 6–10 years (31.7%), 11–15 years (19.2%), and 16 years or above (33.4%). Most teachers are 30–49 years old (80.7%), and about half of them obtain the government’s accredited title (48.5%). Teachers’ educational accomplishment was as follows: 63.8% with a bachelor diploma or higher, 27.0% with associate degree, as well as 9.2% with high school degree or below.

### Measures

Validated items were employed to examine the TAM based on previous studies. The TAM constructs—that is, job relevance, computer self-efficacy, perception of external control, perceived usefulness, perceived ease of use, and behavioral intention—were measured according to Venkatesh and Bala (2008). The measurement of TAM consist of 6 dimensions and 22 items: The job relevance (e.g., “In my job, usage of the system is important,” 3 items), computer self-efficacy (e.g., “I could complete the job using the educational technology if someone showed me how to do it first,” 4 items), perception of external control (e.g., “I have the resources necessary to use the educational technology,” 4 items), perceived usefulness (e.g., “Using the educational technology improves my performance in my job,” 4 items), perceived ease of use (e.g., “In my job, usage of the educational technology is important,” 4 items), and behavioral intention (e.g., “I plan to use this educational technology in the future,” 3 items). In the current study sample, Cronbach’s α of the subscales for job relevance, computer self-efficacy, perception of external control, perceived usefulness, perceived ease of use, and behavioral intention were 0.87, 0.85, 0.83, 0.84, 0.87, and 0.85, respectively.

### Procedures

First, we developed a Chinese version of electronic questionnaire and modified original items according to the situation of preschool teachers’ educational technology use during the COVID-19. Second, a pilot test was conducted among 30 preschool teachers to collect feedback. The e-questionnaire was improved and revised based on these preliminary results. Also, the results show that the time range of e-questionnaire response is 11–17 min. Finally, a convenience sampling technique was employed using Wenjuanxing, a professional online questionnaire collection platform in China. Specifically, preschool teachers were randomly recruited from the National Training Program for Preschool Teachers in China. The teacher agreed to participate voluntarily after receiving information about the research objectives. Researchers send e-questionnaires by sending hyperlinks or QR codes to teachers containing the instructions and a packet of surveys. Teachers can work on the questionnaires by phone or computer at any time in the two weeks. If teachers are disturbed during the investigation, they have opportunities to continue to complete the survey at their convenience.

To ensure the objectivity of the data, we define two submission principles: (a) the same respondent and questionnaires from the same IP address cannot submit the e-questionnaire repeatedly; (b) the answer time should not be less than 10 min. To get participants to answer questions openly and honestly, e-questionnaire were sent directly to teachers via anonymous links. Participants were encouraged to invite their colleagues to participate in the study. Two weeks later, 1,568 e-questionnaires were finished and returned. The research was carried out in accord with the ethical standards in the treatment of human participants and the work was approved by the Institutional Review Board at the study’s home institution.

### Data Analysis

Descriptive and correlation statistics were analyzed in IBM SPSS Statistics 22, and structural equation model (SEM) were conducted using Mplus 8.4. Five variables were regard as covariates in the structural model. We evaluated the fit of the model by the following indicators: The Chi-Square statistic (χ*^2^*), Comparative Fit Index (CFI), Tucker-Lewis Index (TLI), Root-Mean-Square Error of Approximation (RMSEA), and Standardized Root-Mean-Square Residual (SRMR). A Chi-Square statistic of 5 or less is typically considered acceptable (Bollen, 1989). As for the CFI and TLI, estimates are greater than 0.90 exhibiting an acceptable fit (Byrne, 2001; Kline, 2005). The RMSEA and SRMR of 0.08 or less show a good fit to data (Hu and Bentler, 1999).

## Results

### Preliminary Analysis

The descriptive statistics and correlations of all variables are presented in Table 1. All variables are measured by using a 5-point Likert scale, with 3 being the theoretical midpoint. Four variables’ scores fall between 3.35 and 3.72, indicating that preschool teachers provide mid-to-high level on all constructs. Comparing the scores of the six variables, we found that preschool teachers reported the lowest score on perceived ease of use (*M* = 3.35) toward educational technology. The spread of variables’ standard deviation is between 0.69 and 0.77, suggesting a small range of variation. Skewness and Kurtosis values range from 0.12 to 0.25 and from 0.08 to 0.45, respectively. According to the recommended thresholds in the literature, Skewness and Kurtosis’ values in the current study both indicate that all constructs fall within the acceptable range of normality data (Ori et al., 2009).

**TABLE 1:** Correlations and descriptive statistics for the main study variables.

**Constructs**	**1**	**2**	**3**	**4**	**5**	**6**
(1) Job relevance	–					
(2) Computer self-efficacy	0.64***	–				
(3) Perceptions of external control	0.60***	0.78***	–			
(4) Perceived usefulness	0.51***	0.71***	0.56***	−⁣−		
(5) Perceived ease of use	0.60***	0.69***	0.72***	0.69***	–	
(6) Behavioral intention	0.62***	0.76***	0.64***	0.61***	0.56***	–
Mean	3.49	3.55	3.67	3.62	3.35	3.72
Standard deviation	0.77	0.72	0.71	0.73	0.69	0.75
Skewness	–0.03	0.25	–0.02	0.04	–0.12	–0.12
Kurtosis	0.18	0.45	0.34	0.14	0.41	0.08

***p < 0.001 (two-tailed).

As shown in Table 1, correlation values are positively at a significant level of 0.05, ranging from 0.51 to 0.78. The highest correlation coefficient is found between computer self-efficacy and perception of external control (*r* = 0.78, *p* < 0.001).

### Structural Model

**FIGURE 2:**
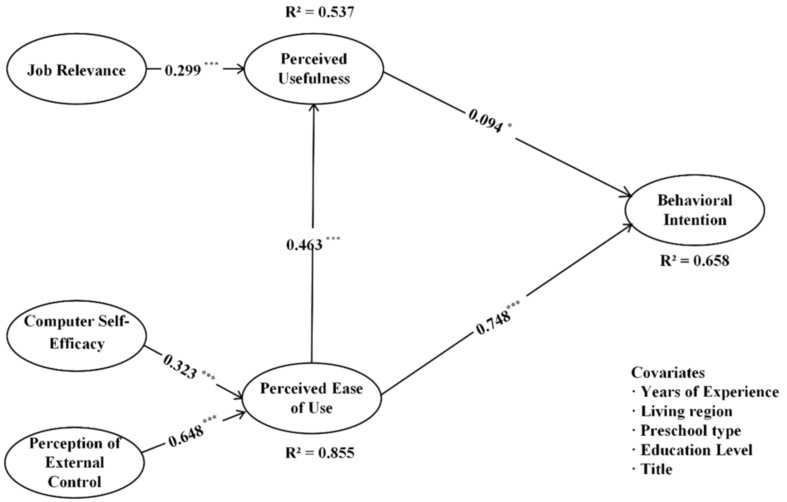
Verified model for behavior intention to use.

Structural equation model was employed using maximum likelihood estimation to evaluate relationships among the latent variables when the measurement models’ validity and reliability were confirmed. All the obtained fit indices of the structure model meet the suggested values in the literature (Hair et al., 2010): χ*^2^* = 3574.63, *df* = 331; CFI = 0.91, TLI = 0.90, SRMR = 0.06, RMSEA = 0.07 with 90% CI [0.07, 0.08]. The path coefficients and their significance are present in Figure 2. To be specific, perceived usefulness and perceived ease of use positively affect behavioral intention (β = 0.094, *p* < 0.05, β = 0.748, *p* < 0.001; respectively), and perceived usefulness is influenced by perceived ease of use (β = 0.463, *p* < 0.001). The positive association was found between job relevance and perceived usefulness (β = 0.299, *p* < 0.001). Also, computer self-efficacy and perception of external control are positively associated with perceived ease of use intention (β = 0.323, *p* < 0.001, β = 0.648, *p* < 0.001; respectively).

Bootstrapping with 2000 resamples was used to assess the significance of the path coefficients. Results indicated that the 95% CI for the direct effect did not include zero, which suggests all of the hypotheses are supported (see Table 2 for details).

**TABLE 2:** Results of the structural model.

**Paths**	**Coefficient**	**95% CI**	**Hypotheses**
H1: Perceived usefulness → Behavioral intention	0.094*	[0.034, 0.173]	Supported
H2: Perceived ease of use→ Behavioral intention	0.748***	[0.845, 0.931]	Supported
H3 Perceived ease of use→ Perceived usefulness	0.463***	[0.175, 0.359]	Supported
H4: Job relevance→ Perceived usefulness	0.299***	[0.398, 0.625]	Supported
H5: Perceptions of external control→ Perceived ease of use	0.323***	[0.209, 0.273]	Supported
H6: Computer self-efficacy→ Perceived ease of use	0.648***	[0.584, 0.639]	Supported

*p < 0.05. ***p < 0.001 (two-tailed).

## Discussion

As a response to the coronavirus, preschools were closed in China from February 2020, which leads to a huge shift from offline to online education. Obviously, online education requires preschool teachers to master information technology. However, it is still unknown whether preschool teachers are willing to accept educational technology. This study applied the adapted TAM to detect Chinese preschool teachers’ acceptance toward educational technology during the COVID-19. Our findings present powerful evidence for the applicability of the adapted TAM under emergency circumstances. Results showed that preschool teachers’ behavioral intention was moderate to high level, which affected by perceived usefulness, perceived ease of use, job relevance, computer self-efficacy, and perception of external control. Specifically, perceived usefulness and perceived ease of use are direct significant predictors of preschool teachers’ behavioral intention. Job relevance is positively associated with perceived usefulness, and computer self-efficacy and perceptions of external control are positively related to perceived ease of use. Our findings create a deeper understanding of the preschool teachers’ acceptance inclination toward educational technology during the COVID-19 epidemic and provide insights into teacher training worldwide, including in China.

This study found that most preschool teachers accepted educational technology during the epidemic as the mean behavioral intention score was above 3, indicating moderate or above agreement. The relatively strong behavioral intentions may be the result of vigorously advocated by the Ministry of Education in China. The Ministry of Education has launched home-based online education and provided online resources and professional training for teachers having access to educational technology (Ministry of Education, 2020). The hard work of the Ministry of Education during the COVID-19 epidemic may have increased the willingness of preschool teachers to use it. In addition, among these variables, the score of preschool teachers’ perceived ease of use is the lowest, which indicates that preschool teachers have difficulty in the actual usage of technology. The most straightforward explanation could be that preschool teachers do not have extensive experience using educational technology. Faced with the sudden onrush of online education during the COVID-19, preschool teachers experienced difficulties in using educational technology. As pointed out in the study of Rafique et al. (2020), the sudden shift of teachers to online education requires the preparation of using platforms, tools and organizing workflows, while the fact is that most teachers do not have mature experience in online education. This may be one of the reasons for the low score of teachers’ perceived ease of use. This not only shows that preschool teachers faced technology challenges in their professional practices (Ertmer and Ottenbreit-Leftwich, 2010), but also provides us with a breakthrough window to enhance preschool teachers’ intention to use educational technology.

Results indicated that the behavioral intention of preschool teachers on educational technology is not only positively affected by perceived ease of use, but also positively affected by perceived usefulness. Our results agree with the existing research examining the TAM (Okumus et al., 2016; Scherer et al., 2019). Perceived usefulness and perceived ease of use are the two fundamental constructs playing a central role in the adoption process of a system (Marangunić and Granić, 2015; Liu et al., 2019). Moreover, from experience, preschool teachers who feel relaxed to using educational technology would like to operate educational technologies. Otherwise, preschool teachers may reject educational technology if it is difficult to use. Also, awareness of the technology usefulness would promote their willingness to use educational technology. In other words, the more useful educational technology is for their work, the more willing preschool teachers are to use it. Compared with the existing research, this study makes outstanding contributions. Although TAM has dominated the research landscape, it has not been focused on preschool teachers under the COVID-19 epidemic. This work at hand provides partial support to literature through testing the importance of perceived ease of use and perceived usefulness for individuals’ behavioral intention using a sample of preschool teachers, a pattern similar to that before the COVID-19 outbreak.

Notably, we found that preschool teachers’ behavioral intention is more strongly influenced by their perceived ease of use instead of perceived usefulness. The surprising result is inconsistent with prior literature including the samples of non-preschool teachers (Scherer et al., 2019). For example, perceived usefulness had the highest influence on teachers’ intention to use information and communication technology in Baydas and Goktas (2016). Our findings mean that preschool teachers tend to focus on the ease of understanding, accepting, and using when they use educational technology. The educational technology’ simplicity and ease of use would enhance preschool teachers’ willingness to use it. A possible explanation could be that preschool teachers lack the ability to use educational technology. On the one hand, although Chinese preschool teachers had been exposed to educational technology before the outbreak of the COVID-19 epidemic, face-to-face communication and teaching dominated their teaching practice. Preschool teachers have few opportunities to use educational technology. On the other hand, pre-service education and post-service training of preschool teachers do not focus on educational technologies’ application. Although perceived ease of use may not be a crucial predictor during forming behavioral intentions at the late stage of technology use (Venkatesh et al., 2003), perceived ease of use is the key factor for beginners. As the epidemic has affected global education, the sudden rise of online education has posed a huge challenge to preschool teachers with limited educational technology ability. If the application of educational technology is free of effort, then preschool teachers would show stronger behavioral intention. Otherwise, preschool teachers would not be willing to apply educational technology.

Besides, consistent with previous studies, perceived ease of use positively impacts perceived usefulness was confirmed in our study. This result is supported by empirical studies (Cigdem and Ozturk, 2016; Rafique et al., 2018). A study of users’ intentions with E-government also found that perceived ease of use has a positive and significant influence on perceived usefulness (Chen and Aklikokou, 2019). Users would emphasize perceived ease of use in shaping perceived usefulness. If preschool teachers feel that educational technologies are challenging to apply, then they will hold that educational technologies will have little effect on their work. That is, preschool teachers will believe the technology is helpful and useful during the COVID-19 pandemic when they perceive educational technology is easy to apply.

Also, our current study supported job relevance as an influencing factor of perceived usefulness. When technology facilitates individual work and improves work efficiency in an accurate, understandable, and effective way (Speier et al., 2003), preschool teachers are more likely to experience greater job relevance of the technology, which eventually promotes teachers’ perceived usefulness. What’s more, preschool teachers’ perceived ease of use is predicted positively by both perception of external control and computer self-efficacy. Our results are supported by previous literature (Son et al., 2012; Ros et al., 2015). A study was conducted on company staff and found that self-efficacy has a direct impact on the perceived ease of use of big data tools (Okcu et al., 2019). Surprisingly, compared with computer self-efficacy, perception of external control influences perceived ease of use in a significantly greater way, which is consistent with previous studies (Venkatesh and Bala, 2008). When preschool teachers receive more organizational resources and support during the COVID-19 pandemic (Ngai et al., 2007), such as equipment and information about using educational technology, they would perceive more strongly that technologies are easier to apply. Therefore, with a growing perception of external control in using technology, their use intentions would be correspondingly improved.

The TAM’s broad applicability is demonstrated by numerous studies about a diverse series of technologies and users. However, the unique contribution of our work is to identify behavioral intentions’ potential determinants based on preschool teachers who have not prepared well in technology knowledge and skills during the COVID-19 pandemic. The results indicated that the adapted TAM fits well. This study provides further evidence that the adapted TAM is suitable for measuring educational technology’s acceptance intention to in the normal and emergency period. Significantly, preschool teachers’ perceived usefulness only shows a weak direct impact on using intention to educational technologies, whereas perceived ease of use shows a directly strong influence upon using intention. On the other hand, the impact of preschool teachers’ perceived external control on their perceived ease of use is stronger than that of computer self-efficacy.

## Implications

Despite the increasing interest in incorporating technology into educational settings, research on investigating the technology acceptance is still absent in preschool education field, especially during the COVID-19 crisis. Our study could help technology acceptance research in both theory and practice. Theoretically, our study enriches the existing empirical research by testing the adapted TAM’s applicability for preschool teachers under a public health emergency period. Existing literature and our work at hand have found that TAM model has applicability and robustness in both general and special situations. Secondly, we also extended the samples of technology acceptance research from western teachers to Eastern ones, namely, samples of preschool teachers from developing countries, so that the Eastern and Western research findings can be better discussed in the same discourse framework. Third, the adapted the TAM with perceived usefulness, perceived ease of use, job relevance, perception of external control and computer self-efficacy reveals the factors that could influence preschool teachers’ technology adoption during the COVID-19 pandemic.

Practically, our findings could provide suggestions for educational technology system developers, preschool managers, and policy-makers. Improving the convenience of the educational technology can be considered when technology developers design the system (Yang and Wang, 2019). Technology developers could simplify the operation of educational technologies based on preschool teachers’ feedback and provide comprehensible instructions or videos. For preschool managers, basic educational technology training, learning community, and available technology professionals could be effective support ways to make the teachers control educational technology, and thus improve the preschool teachers’ behavioral intention. As for policy-makers, efforts to integrate educational technology into teacher education and preservice teacher education programs are necessary (Perkmen et al., 2016). In pre-service education, educational technical knowledge and skills can be incorporated into the curriculum for student teachers majoring in preschool education. For in-service teachers, education departments can consider developing educational technology training programs to improve the ability of preschool teachers’ educational technology competence. In addition, the government, especially the Ministry of Education, could update the national professional standards for preschool teachers requiring them to master the necessary educational technical knowledge and skills.

## Limitations and Conclusion

There are several limitations in the current work. First, our findings are obtained from cross-sectional data with preschool teachers’ self-reports. Preschool teachers’ perception and intention of using educational technology may vary with the using duration. The longitudinal design could be employed to examine the change of preschool teachers’ behavioral intention and its determinants. Second, the data in this study were obtained through e-questionnaires when government has taken social isolation measures to prevent the spread of the COVID-19 epidemic. The results may be exaggerated by social effects. Observations and face-to face interviews can be used to obtain data objectively and results in the future. Also, observations and interviews can also be used to obtain data objectively and comprehensively. Third, notice should be taken when replicating and verifying the results to a large sample. Considering preschool teachers with different backgrounds, future research could use teachers from different regions or even different countries. Fourth, our current results are based on a sample of preschool teachers. Hence, the present results might not be the same for the other teachers from primary and secondary schools, high schools, universities, and vocational schools. Given the differences in work content and experience, future research could consider recruiting teachers from other educational stages. Last, the present study only tested job relevance as predictors for perceived usefulness, and computer self-efficacy and perception of external control as factors for perceived ease of use. There may be more alternative determinants, such as subjective norm, computer anxiety, and output quality, which merit future research (Legris et al., 2003).

Overall, despite the above limitations, this study is the first to analyze, employing the adapted TAM, preschool teachers’ technology acceptance intention and its influencing factors during the epidemic. Although the TAM is dominant in literature, previous studies have not focused on preschool teachers affected by the global pandemic. In the context of the COVID-19 epidemic, our work at hand examined the adapted TAM model in the data from preschool teachers. The results indicate that preschool teachers’ behavioral intention of educational technology is in the middle level, and preschool teachers’ behavioral intention was directly or indirectly affected by perceived usefulness, perceived ease of use, job relevance, computer self-efficacy, and perception of external control. Our work contributes to the literature by focusing on the behavioral intention of Chinese preschool teachers, who have experienced online teaching at home for about a semester under the COVID-19. Our research contributes to finding out potential determinants impacting preschool teachers’ intention to apply educational technologies. The findings are helpful to develop targeted practical measures to improve preschool teachers’ technical acceptance intention and thus improve the quality of online education.

## Data Availability Statement

The raw data supporting the conclusion of this article will be made available by the authors, without undue reservation.

## Ethics Statement

The studies involving human participants were reviewed and approved by Institute of Early Childhood Education, Faculty of Education, Beijing Normal University. The patients/participants provided their written informed consent to participate in this study. Written informed consent was obtained from the individual(s) for the publication of any potentially identifiable images or data included in this article.

## Author Contributions

XH and MZ designed the research and wrote the manuscript. QL was mainly responsible for the sections “Introduction” and “Implications.” All authors contributed to the article and approved the submitted version.

## Conflict of Interest

The authors declare that the research was conducted in the absence of any commercial or financial relationships that could be construed as a potential conflict of interest.
